# Immunogenicity of Varicella-Zoster Virus Glycoprotein E Formulated with Lipid Nanoparticles and Nucleic Immunostimulators in Mice

**DOI:** 10.3390/vaccines9040310

**Published:** 2021-03-25

**Authors:** Han Cao, Yunfei Wang, Ning Luan, Cunbao Liu

**Affiliations:** Institute of Medical Biology, Chinese Academy of Medical Sciences and Peking Union Medical College, Kunming 650118, China; caohan@imbcams.com.cn (H.C.); wangyf@imbcams.com.cn (Y.W.); luanning@imbcams.com.cn (N.L.)

**Keywords:** herpesvirus 3, human, varicella, chickenpox, herpes zoster, shingles, humoral immunity, cell-mediated immunity, nucleic acid immunostimulator, Poly I:C (polyinosinic-polycytidylic acid), CpG ODN (CpG oligodeoxynucleotide), lipid nanoparticle

## Abstract

Theoretically, the subunit herpes zoster vaccine Shingrix^TM^ could be used as a varicella vaccine that avoids the risk of developing shingles from vaccination, but bedside mixing strategies and the limited supply of the adjuvant component QS21 have made its application economically impracticable. With lipid nanoparticles (LNPs) that were approved by the FDA as vectors for severe acute respiratory syndrome coronavirus 2 vaccines, we designed a series of vaccines efficiently encapsulated with varicella-zoster virus glycoprotein E (VZV-gE) and nucleic acids including polyinosinic-polycytidylic acid (Poly I:C) and the natural phosphodiester CpG oligodeoxynucleotide (CpG ODN), which was approved by the FDA as an immunostimulator in a hepatitis B vaccine. Preclinical trial in mice showed that these LNP vaccines could induce VZV-gE IgG titers more than 16 times those induced by an alum adjuvant, and immunized serum could block in vitro infection completely at a dilution of 1:80, which indicated potential as a varicella vaccine. The magnitude of the cell-mediated immunity induced was generally more than 10 times that induced by the alum adjuvant, indicating potential as a zoster vaccine. These results showed that immunostimulatory nucleic acids together with LNPs have promise as safe and economical varicella and zoster vaccine candidates.

## 1. Introduction

As its name indicates, varicella-zoster virus (VZV) causes two distinct diseases, i.e., varicella/chickenpox upon primary infection and zoster/shingles when latent viruses in the sensory ganglia reactivate [1]. In fact, nearly everyone comes in to contact with this virus before adulthood and shingles affects one in three people during their lifetime [2]. While attenuated strains (e.g., the Oka strain) have been used worldwide as varicella vaccines at a dose of 1000–10,000 plaque-forming units (PFU) since approval by the FDA in 1995, they may remain in the sensory ganglia and reactivate similar to the corresponding wild-type strains, causing herpes zoster in immunosenescent (e.g., older people) and immunocompromised populations (e.g., HIV carriers and cancer chemotherapy patients), which may result in postherpetic neuralgia that lasts for weeks to years without effective pain relievers available [3,4,5,6,7,8,9].

Cell-mediated immunity (CMI) rather than humoral immune responses has been indicated to play a key role in restricting latent VZV and preventing zoster [10,11,12,13,14]. On the basis of this information, the following two forms of zoster vaccines that boost pre-existing cellular immune responses caused by primary exposure or varicella vaccination are available on the market: a single subcutaneous dose of an attenuated virus as high as 20,000 PFU in ZOSTVAX^®^ (developed in 2005 by Merk & Co., Inc., Kenilworth, NJ, USA) or two intramuscular doses of the subunit vaccine Shingrix^TM^ (developed in 2017 by Glaxo Smith Kline (GSK), Rockville, MD, USA) which contains the extracellular domain of VZV glycoprotein E (gE) and the AS01B adjuvant system. In addition to the strict conditions required for manufacturing and maintaining the necessary high titers of ZOSTVAX^®^, the efficacy of this vaccine declines from 70% in people aged 50–59 to less than 38% in people older than 70 [15,16]. In contrast, the protection rates of Shingrix^TM^ are higher than 90% in all of the age groups tested, including people older than 80, yet bedside mixing strategies and the limited supply of its adjuvant component QS21 have made Shingrix^TM^ very expensive (approximately 150–200 USD per dose) [17,18,19,20,21,22].

In our previous reports, we proved that encapsulation of economical nucleic acid immunostimulators including polyinosinic-polycytidylic acid (Poly I:C) and the natural phosphodiester CpG oligodeoxynucleotide (CpG ODN) into poly(lactic-co-glycolic acid) (PLGA)-based nanoparticles showed excellent efficacy in therapeutic TC-1-grafted tumor models, in which CMI responses play decisive roles [23,24]. Although this economical adjuvant system also showed potential roles in VZV-gE CMI responses, the application of PLGA carriers is approved by the FDA for only chemical medicines, not vaccines. In addition, the encapsulation efficiency of nucleic acids by PLGA is less than 30% through the double-emulsion (w/ow) solvent evaporation method, which needs to be improved [25,26].

Because of its high contagiousness, morbidity, and mortality characteristics, severe acute respiratory syndrome coronavirus 2 (SARS-CoV-2) has spread worldwide within months. Correspondingly, a new form of vaccine based on ionizable lipids, which were once designed as carriers for siRNA therapy for cancers, was adopted as an mRNA carrier and approved by the FDA within 1 year to prevent coronavirus disease 2019 (COVID-19) [27,28]. These lipid nanoparticles (LNPs) not only show good safety but also excellent nucleic acid encapsulation efficiency and potency to facilitate cellular uptake and endosomal escape, which are quite helpful in inducing antigen-specific CMI responses that play a key role in restricting latent VZV and preventing zoster, as mentioned above [29,30]. On the basis of this background information, we produced a series of potential subunit vaccines based on LNPs, tested the encapsulation efficiencies of VZV-gE and nucleic acid immunostimulators, and evaluated the immunogenicity of the vaccines in mice and their potency as VZV vaccines.

## 2. Materials and Methods

### 2.1. Vaccines

The compositions of the designed vaccines are shown in Table 1.

LNP vaccines were prepared using a modified procedure previously described for mRNA vaccines [31,32,33]. Briefly, lipids (from AVT Pharmaceutical Technology Co., Ltd., Shanghai, China) were dissolved in ethanol at molar ratios of 50:10:37.5:2.5 (MC3/DSPC/cholesterol/DMG-PEG2000). The lipid mixtures were combined with 100 mM citrate buffer (pH 4.0) containing the extracellular domain of gE expressed in Chinese hamster ovary cells (supplied by AtaGenix Laboratory Co., Ltd., Wuhan, China), high-performance liquid chromatography-grade phosphodiester CpG ODN including BW006 (5′-tcg acg ttc gtc gtt cgt cgt tc-3′) and 2395 (5′-tcg tcg ttt tcg gcg c:gc gcc g-3′) (supplied by Sangon Biotech Co., Ltd., Shanghai, China), and low-molecular-weight Poly I:C (InvivoGen, Inc. San Diego, CA, USA) at a ratio of 3:1 with a microfluidic mixer (Precision Nanosystems, Inc., Vancouver, BC, Canada). Formulations were dialyzed against PBS, concentrated with a centrifugal filtration tube (Millipore), passed through a 0.22 μm syringe filter (PALL), and stored at 4 °C until use. Particle sizes were tested with a Zetasizer Nano ZS particle size analyzer (Malvern Panalytical, Malvern, UK). Loaded gE was detected with a bicinchoninic acid protein assay kit (Beyotime, Shanghai, China) and encapsulation efficiency was calculated as the amount of loaded gE detected as compared with the initial amount of gE input in citrate buffer. Loaded nucleic acids were detected with the Quant-iT OliGreen ssDNA Reagent Kit (Thermo Fisher, Eugene, OR, USA) and encapsulation efficiency was calculated as the amount of loaded nucleic acids detected as compared with the amount of initial nucleic acids input in citrate buffer.

### 2.2. Preclinical Trial in Mice

Six-week-old female specific pathogen-free (SPF) C57BL/6N mice (15–18 g) were supplied by Vital River Laboratory Animal Technology Ltd. (Beijing, China), randomly divided into 9 groups with 6 mice in each group (*N* = 6), maintained under SPF conditions and housed with free access to food and water at the Central Animal Services of the Institute of Medical Biology, Chinese Academy of Medical Sciences and Peking Union Medical College (IMB, CAMS). The mice were immunized intramuscularly in the thigh muscle three times with 50 μL of immunogen at 2-week intervals. Blood samples (via cardiac puncture) and spleens were collected 2 weeks after the final immunization. After clotting at 4 °C overnight, serum was collected after centrifugation at 3500 rpm for 20 min.

### 2.3. Enzyme-Linked Immunosorbent Assay (ELISA) for gE-Specific Antibodies

gE (2 μg/mL) was used to coat 96-well plates (Corning) at 4 °C overnight. After blocking with 5% (*w*/*v*) skim milk at 37 °C for 1 h, the plates were incubated with serial dilutions of mouse sera at 37 °C for 1 h. Bound antibodies were detected with goat anti-mouse IgG-horseradish peroxidase (HRP) conjugate (1:5000, Bio-Rad, Hercules, CA, USA) as a secondary antibody. Ten minutes after the addition of the substrate 3,3′,5,5′-tetramethylbenzidine (BD), 1 mol/L phosphoric acid was added to terminate the reaction. The absorbance at 450 nm was detected with a spectrophotometer (BioTek Instruments, Inc., Winooski, VT, USA). IgG titers were defined as end-point dilutions showing cutoff signals above OD450 = 0.1, and IgG titers lower than 100 were defined as 100 for calculations.

### 2.4. Fluorescence-Based Plaque Reduction Neutralization Assay

Live attenuated VZV (Changchun BCHT Biotechnology Co., Ltd., Changchun, China) was incubated with mouse sera at given dilutions at 37 °C for 1 h and added to monolayer MRC-5 cells (human fetal lung fibroblasts, Conservation Genetics CAS Kunming Cell Bank, Kunming, China) in 96-well plates (Corning) for 1 h, and then the plates were washed with Dulbecco’s modified Eagle’s medium (DMEM, Thermo Fisher). The cells were incubated with DMEM without fetal bovine serum (FBS) for 16 h at 37 °C in 5% CO_2_. After washing with PBS, the plates were fixed with precooled 80% (*v*/*v*) acetone at −20 °C for 10 min and blocked with 2% (*w*/*v*) skim milk at 37 °C for 1 h. After washing 3 times with PBS, in-house rabbit anti-VZV antiserum (1:250) was added and incubated at 37 °C for 1 h. The bound antibodies were detected with fluorescein isothiocyanate (FITC)-labeled goat anti-rabbit IgG antibodies (1:100, Cayman Chemical Company, Ann Arbor, MI, USA) at 37 °C for 1 h. Plates were imaged with a Cytation 1 imaging reader (BioTek Instruments, Inc., Winooski, VT, USA) [34].

### 2.5. Enzyme-Linked Immunospot (ELISPOT) Assay

Spleens were dispersed with a 70 μm cell strainer (NEST, Wuxi, China). After red blood cell lysis with ammonium chloride potassium buffer, splenocytes were collected by centrifugation at 1800 rpm for 5 min, the number of cells was calculated, and the cells were suspended in Roswell Park Memorial Institute (RPMI, Thermo Fisher) 1640 medium supplemented with 10% (*v*/*v*) FBS (Biological Industries, Cromwell, CT, USA) and penicillin-streptomycin (Thermo Fisher) at a final concentration of 3 × 10^6^ cells/mL. Then, 100 μL of cells were added to each well of a 96-well plate (Corning Inc., Corning, NY, USA) for further analysis with an ELISPOT assay kit (BD), according to the manufacturer’s protocol. gE and pooled peptides (purity ≥ 95%, synthesized by GL Biochem Co., Ltd. Shanghai, China) at 10 μg/mL were both selected to stimulate gE-specific T cell responses by incubation of cells with protein/peptides overnight. Spots were counted with an ELISPOT reader system (Autoimmun Diagnostika GmbH, Strassberg, Germany) after immunoimaging [26].

### 2.6. Statistical Analysis

gE concentrations and encapsulation efficiency were compared with an unpaired t-test. Nucleic acid concentrations, encapsulation efficiency, IgG titers, and ELISPOT numbers were compared using one-way analysis of variance (ANOVA) followed by Dunnett’s multiple comparisons test with the LNP-BW006+2395-gE group as the control. Diameters and polydispersity index (PDI) results were compared using one-way ANOVA followed by Tukey’s multiple comparisons test (GraphPad Prism 7.0 software, GraphPad Software Inc., La Jolla, CA, USA) (* *p* < 0.05; ** *p* < 0.01; *** *p* < 0.001; **** *p* < 0.0001; ns, no significant difference).

## 3. Results

### 3.1. LNPs Efficiently Encapsulated gE and Nucleic Acid Immunostimulators

**Figure 1:**
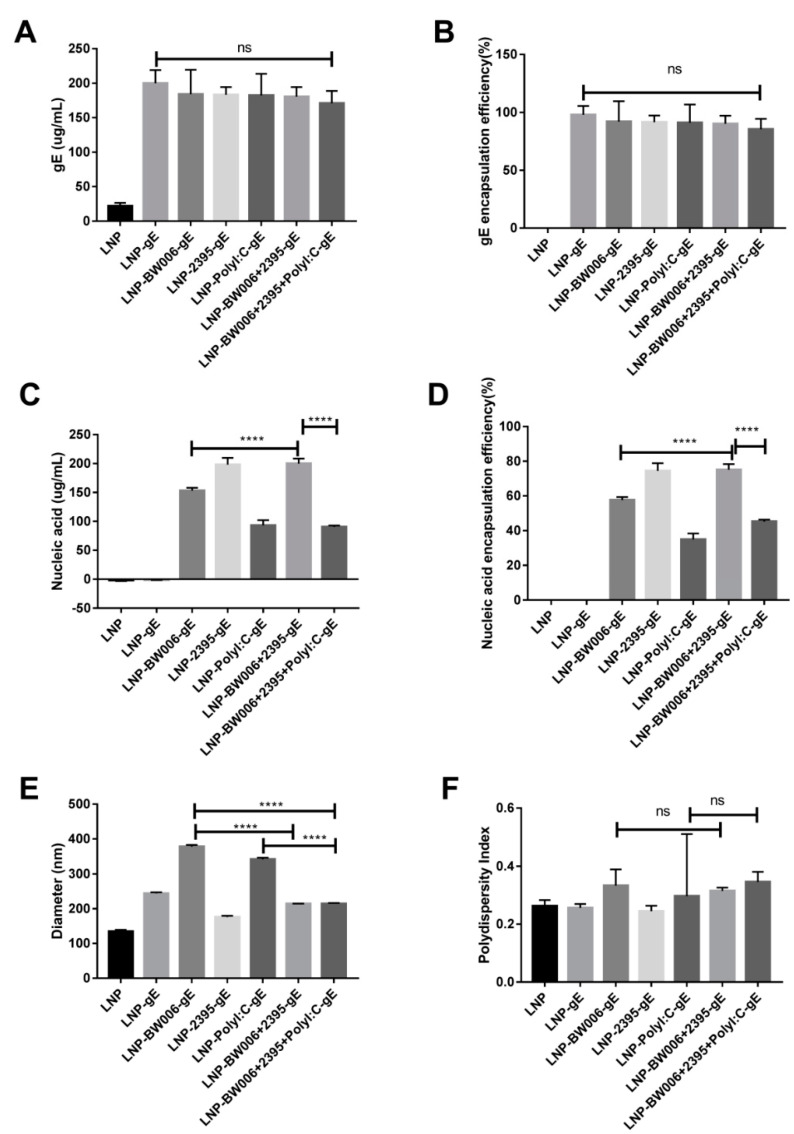
Characterization of LNP vaccines. (**A**) gE concentration; (**B**) gE encapsulation efficiency; (**C**) Nucleic acid immunostimulator concentration; (**D**) Nucleic acid immunostimulator encapsulation efficiency; (**E**) Diameters tested by a size analyzer; (**F**) Polydispersity index of LNPs. **** *p* < 0.0001. ns, no significant difference.

As shown in Table 1, 10 μg gE were designed for each dose of vaccine. When 300 μg gE was added to 1.5 mL citrate buffer as the raw materials for 20 doses of LNP vaccines, the encapsulation efficiency was nearly 100% (group LNP-gE in Table 2 and Figure 1A,B), which is 50% more than we expected. Coencapsulation of nucleic acid immunostimulators showed limited interference with the gE loading efficiency except for in the LNP-BW006+2395+Poly I:C-gE group (85% in Figure 1B), which had the lowest total nucleic acid input among all of the groups (Table 1), implying that nucleic acid characteristics instead of nucleic acid loading limits may influence the loading efficiency of gE in LNPs.

According to our previous experiences on the potency of nucleic acids to stimulate adaptive immune responses and the nucleic acid loading efficiency of ionizable lipids, up to 12.5 μg nucleic acids were designed for each dose of vaccine (Table 1) [24,35]. When 400 μg nucleic acid immunostimulators were added to 1.5 mL citrate buffer with 300 μg gE as the raw materials for 20 doses of LNP vaccines, the encapsulation efficiency varied (Table 2 and Figure 1C,D). While CpG ODN 2395 alone (vaccine LNP-2395-gE) showed an encapsulation efficiency of 74.4%, CpG ODN BW006 alone (vaccine LNP-BW006-gE) showed a lower encapsulation efficiency of 57.6%, and Poly I:C alone (vaccine LNP-Poly I:C-gE) showed the lowest encapsulation efficiency of 35%, which is lower than the mRNA encapsulation efficiency reported (69–100%) [35]. Interestingly, CpG ODN 2395 seemed to be helpful for elevating the encapsulation efficiency of CpG ODN BW006, as the coencapsulation efficiency of CpG ODN 2395 and CpG ODN BW006 (vaccine LNP-BW006+2395-gE) was 75.1%, which was much higher (*p* < 0.0001) than the encapsulation efficiency of CpG ODN BW006 alone (vaccine LNP-BW006-gE, 57.6%). This ability seemed to be weakened by the presence of Poly I:C, as the encapsulation efficiency of the LNP-BW006+2395+Poly I:C-gE group was only 45.2%. Because the total nucleic acid input was 375 μg instead of 400 μg in this group, we also attributed this decreased loading efficiency to nucleic acid characteristics instead of nucleic acid loading limits, similar to the conclusion for gE.

While CpG ODN 2395 condensed the diameters of LNPs (214.4 nm for LNP-BW006+2395-gE versus 377.7 nm for LNP-BW006-gE, 214.2 nm for LNP-BW006+2395+Poly I:C-gE versus 341.6 nm for LNP-Poly I:C-gE and 377.7 nm for LNP-BW006-gE) (Figure 1E) and showed some tendency toward uniform LNPs (LNP-BW006+2395-gE versus LNP-BW006-gE, LNP-BW006+2395+Poly I:C-gE versus LNP-Poly I:C-gE) (Figure 1F), only the polydispersity index (PDI, a measure of the heterogeneity of a sample based on size) showed a slight reverse tendency with nucleic acid immunostimulator encapsulation efficiencies.

### 3.2. LNP-Encapsulated gE and Nucleic Acid Immunostimulators Induced Potent Humoral Immune Responses

**Figure 2:**
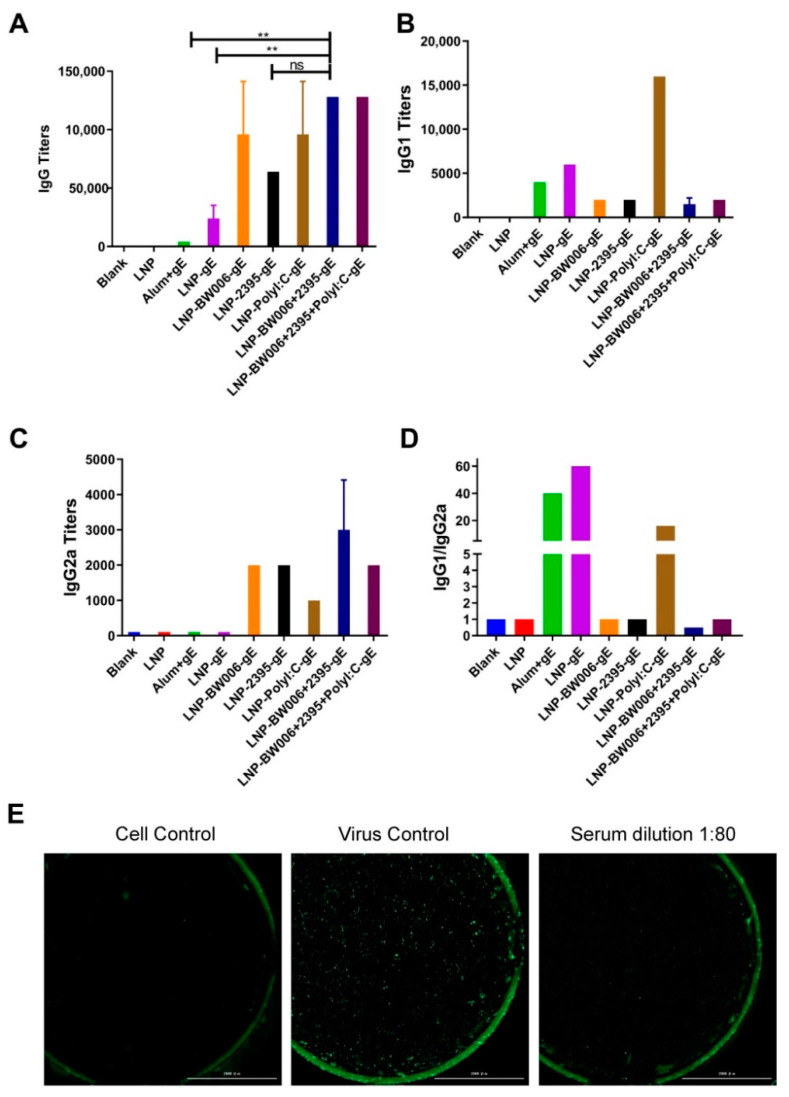
Humoral immune responses. (**A**) gE-specific IgG titers; (**B**) gE-specific IgG1 titers; (**C**) gE-specific IgG2a titers; (**D**) IgG1/IgG2a ratios; (**E**) Neutralizing effects of serum from LNP-BW006+2395-gE-immunized mice. Scale bar, 2000 μm. ** *p* < 0.01. ns, no significant difference.

LNPs with encapsulated gE alone (LNP-gE) induced six times more gE-specific IgG antibodies than the alum adjuvant (24,000 versus 4000, Figure 2A). Coencapsulation of nucleic acid immunostimulators by LNPs further elevated gE-specific IgG antibody levels (all above 64,000). Among the immunostimulators, CpG ODN 2395 seemed to be less potent than CpG ODN BW006 in inducing humoral responses considering its higher encapsulation efficiency (64,000 IgG titers and 74.4% encapsulation for LNP-2395-gE versus 96,000 IgG titers and 57.6% encapsulation for LNP-BW006-gE). Poly I:C should be the most potent immunostimulator for inducing humoral responses because its encapsulation rate in LNP-Poly I:C-gE was approximately 60% of that of CpG ODN W006 in LNP-BW006-gE, yet they induced the same IgG titers (96,000 for both). This potency was confirmed in the LNP-BW006+2395+Poly I:C-gE group, which contained the lowest amount of nucleic acid immunostimulators but induced the highest IgG tiers (128,000), which could be defined as synergistic effects [23]. Interestingly, this phenomenon was also observed between CpG ODN BW006 and CpG ODN 2395, which induced higher IgG titers (128,000 for LNP-BW006+2395-gE) than CpG ODN BW006 alone at the same concentration (96,000 for LNP-BW006-gE).

The IgG1-to-IgG2a titer ratio was calculated to evaluate the Th1-Th1 balance, with the Th1 responses standing for more potent cellular mediated immunity that was better suited to control latent VZV [36,37]. While all of the immunogens containing gE induced obvious IgG1 subtype antibodies (Figure 2B), only LNPs with nucleic acid immunostimulators induced obvious IgG2a subtype antibodies (Figure 2C). Among these LNP formulations, LNP-Poly I:C-gE induced much higher IgG1 antibody titers with an IgG1-to-IgG2a ratio of 16, which indicated a Th2-dominant humoral immune response similar to that induced by the alum adjuvant and LNP alone (Figure 2D) [37,38,39]. Correspondingly, the presence of CpG ODNs was accompanied by more balanced Th1-Th2 responses, and coencapsulation of CpG ODN BW006 and 2395 (LNP-BW006+2395-gE) showed more Th1-biased potency, which was indicated by the fact that the IgG1-to-IgG2a ratio in this group was the only one with a value less than 1. Notably, coencapsulation of CpG ODNs could balance the powerful Th2-dominant humoral immune response induced by Poly I:C, which showed an IgG1-to-IgG2a ratio of 1 instead of 16, i.e., the ratio for Poly I:C alone.

A representative image of a VZV neutralization assay is shown in Figure 2E. At a dilution of 1:80, immunized serum from the LNP-BW006+2395-gE group blocked nearly all infection of MRC-5 cells by live attenuated VZV strains, which showed the potency of these LNP vaccines as varicella vaccines.

### 3.3. LNP-Encapsulated gE and Nucleic Acid Immunostimulators Induced Potent CMI

**Figure 3:**
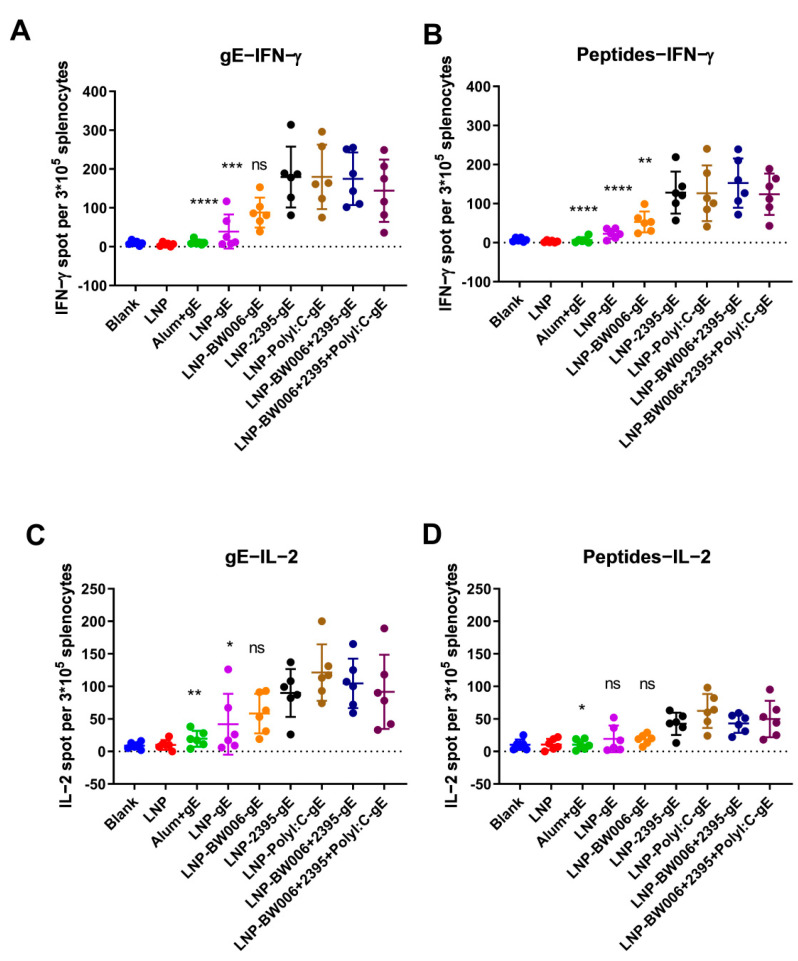
Enzyme-linked immunospot (ELISPOT) assay performed with splenocytes. (**A**) IFN-γ-producing splenocytes after gE stimulation; (**B**) IFN-γ-producing splenocytes after pooled peptide stimulation; (**C**) IL-2-producing splenocytes after gE stimulation; (**D**) IL-2-producing splenocytes after pooled peptide stimulation. * *p* < 0.05, ** *p* < 0.01, *** *p* < 0.001, **** *p* < 0.0001. ns, no significant difference. ELISPOT numbers were compared using one-way analysis of variance (ANOVA) followed by Dunnett’s multiple comparisons test with the LNP-BW006+2395-gE group used as the control.

To verify the CMI responses indirectly suggested by the IgG1-to-IgG2a ratios in Figure 2D, gE-specific IFN-γ- and IL-2-producing splenocytes were detected by ELISPOT. In each group of mice immunized with the same vaccine, incubation of splenocytes with either protein gE (Figure 3A) or peptides from gE (Figure 3B) at the concentration of 10 μg/mL both showed similar tendency of IFN-γ-producing cells as compared with other groups. At the same time, gE are more potent than the peptide pools we selected for stimulation, which showed as higher numbers of IFN-γ-producing cells as compared with peptides stimulated mice immunized with the same vaccine formulations. Notably, while CpG ODN BW006 was more potent in inducing humoral immune responses than CpG ODN 2395 (LNP-BW006-gE versus LNP-2395-gE in Figure 2A), it showed similar or lower potency in inducing IFN-γ-producing splenocytes than LNP-2395-gE (Figure 3A,B), even when its lower encapsulation efficiency was considered (57.6% for LNP-BW006-gE and 74.4% for LNP-2395-gE in Figure 1D). In contrast to CpG ODN BW006, Poly I:C showed higher potency in inducing not only humoral immune responses (LNP-Poly I:C-gE in Figure 2A) but also IFN-γ-producing splenocytes (LNP-Poly I:C-gE in Figure 3A,B), especially when its lower encapsulation efficiency was considered (35% for LNP-Poly I:C-gE in Figure 1D). Synergistic effects on the stimulation of IFN-γ-producing splenocytes were detected between CpG ODN BW006 and 2395 (LNP-BW006+2395-gE versus LNP-BW006-gE and LNP-2395-gE in Figure 3A,B), considering the lower potency of IFN-γ-producing splenocyte induction by CpG ODN BW006. These synergistic effects were also identified between CpG ODN BW006, CpG ODN 2395, and Poly I:C in LNP-BW006+2395+Poly I:C-gE considering their lower coencapsulation efficiencies, as we concluded for the humoral immune response.

The above patterns were also observed for the stimulation of IL-2-producing splenocytes, as shown in Figure 3C,D. Compared with LNP vaccines with encapsulated nucleic acid immunostimulators, LNP vaccines alone (LNP-gE) and the alum adjuvant induced very low CMI according to our ELISPOT analyses of both IFN-γ and IL-2, which suggested the potency of LNP-encapsulated gE and nucleic acid immunostimulators as zoster vaccines.

## 4. Discussion

Because of the highly contagious character of VZV, nearly everyone comes into contact with this virus before adulthood. Live attenuated vaccines (e.g., the Oka strain) have been used worldwide to prevent primary infection, which causes varicella/chickenpox. Unfortunately, the live attenuated strain used in vaccines may lurk in the sensory ganglia and reactive as shingles/zoster similar to wild-type strains [2,4,5]. Subunit vaccines may be chosen as substitutes for the live attenuated varicella vaccines to prevent both chickenpox and the risk of shingles that comes from vaccination. The gE protein is a conserved glycoprotein essential for the replication and transmission of VZV [40,41,42]. The potential neutralization and T cell epitopes of gE make it an ideal target as a subunit vaccine antigen; however, its immunogenicity should be further strengthened by appropriate adjuvants [40,41,43,44]. Fortunately, gE was efficiently loaded into LNPs that were developed for use as nucleic acid medicine/vaccine vectors (Figure 1A,B). When coencapsulated with nucleic acid immunostimulators in LNPs, gE induced specific IgG titers that were more than 16 times those induced by the alum adjuvant. Serum from immunized mice with an average IgG titer of 64,000 could completely block infection with the Oka VZV strain in vitro at a dilution of 1:80 (Figure 2E), which indicated promise as a safe varicella vaccine.

For zoster vaccines, CMI instead of humoral immune responses plays a key role in restricting latent VZV, which is vital for zoster vaccine efficacy [11]. Though live attenuated VZV (i.e., ZOSTVAX^®^) can boost existing CMI to a certain extent, the difficulties related to virus purification, strict conditions required during transportation, and rapidly declining protective rate in people older than 70 still need to be solved. On the basis of gE and the novel adjuvant system AS01B, Shingrix^TM^ has produced approximately 10 times stronger gE-specific CMI than ZOSTVAX^®^, which is consistent with the higher protection rate of Shingrix™, especially in people older than 70 or 80 [36]. Unfortunately, one of the key components of the adjuvant AS01B (i.e., QS21) is a polysaccharide mixture that cannot be synthesized; it can only be extracted from the bark of *Quillaja saponaria* [45]. The limited distributions of *Q. saponaria* around the globe and strict quality control during the extraction processes of QS21 have made ShingrixT^M^ very expensive (approximately 150–200 USD per dose).

With LNP-encapsulated gE and nucleic acid immunostimulators including Poly I:C and the natural phosphodiester CpG ODN, which can both be produced economically on a large scale, ELISPOT studies have shown that LNP vaccines, except for LNP-BW006-gE, all induced CMI responses above 10 times stronger than those induced by the alum adjuvant (Figure 3). According to a flow cytometry analysis reported in preclinical studies of Shingrix^TM^ performed with mice, AS01B can induce seven-fold increases in CMI specific for gE as compared with an alum adjuvant [46]. According to previous studies on the CMI response based on gE and adjuvant systems including CIA09A, gE-specific ELISPOT tests correlate well with gE-specific cytokine-producing CD4+ and CD8+ T cell frequencies, although Th1 CD4+ cells are adopted more frequently than CD8+ T cells as good indicators for the evaluation of zoster vaccines in animal and clinical experiments [26,47]. The aforementioned CMI effects induced by both AS01B and CIA09A to target gE were tested in VZV-primed animal models, while our tests did not adopt this model due to the lack of high-quality Oka strain-based vaccines with high titers, such as Varilrix from GSK and Zostavax from Merck. In fact, no pathological animal models are available for zoster vaccine studies, which may be partly attributed to the special skin structure of humans as compared with that of existing experimental animals, including mice and nonhuman primates. Primed VZV will neither become latent nor reactivate in mice, although replication may be probable according to reports on the detection of viral DNA 1 month after inoculation [48,49]. Compared with two intramuscular doses of the subunit vaccine or mRNA vaccine after VZV priming, the first dose of our three intramuscular injections might cause a comparably lower base level of VZV-specific immunity, but the effective induction of CMI by these LNP vaccines implied comparable or perhaps better potency than that observed in VZV-primed models, though a parallel study with the same immunization procedure should reflect these CMI responses more directly, and we will compare efficacies once these other formulations, such as Shingrix^TM^, are available [2,46].

Considering their low encapsulation efficiency and strong but Th-2-balanced induced immune responses, adjuvant formulations without Poly I:C should be tested further (Figure 2D). In fact, synergistic effects on humoral responses and CMI that were comparable to those induced with Poly I:C were achieved when CpG ODN BW006 (class B CpG ODNs that are helpful to enhance humoral immunity) and CpG 2395 (class C CpG ODNs that are helpful to enhance both humoral immunity and cellular immunity) were coencapsulated (i.e., LNP-BW006+2395-gE), with a higher encapsulation efficiency (75%), more condensed size (diameter = 214.4 nm), and more uniform shapes (PDI = 0.3) [50,51]. In addition, while CpG ODN was approved in 2017 by the FDA as an adjuvant for the human hepatitis B virus vaccine HEPLISAV-B^TM^ (Dynavax, Emeryville, CA, USA), Poly I:C has been used only as an antiviral medication in China, and the only clinical trial evaluating its application in vaccines by the FDA involves it as an adjuvant for rabies vaccines, which recently completed phase II clinical trial testing [52,53].

While the physical characteristics (including diameters and PDI) and components (including gE and nucleic acid contents) were stable when stored at 4 °C during our study, which is a priority compared with LNP-based mRNA vaccines designed for VZV that are stable at −20 °C at present, longer stability at 4 °C still needs to be tested [2]. Interestingly, while aggregates formed during both the freeze-thaw cycles and the lyophilization-reconstitution process of LNP-based siRNA vaccines, the addition of lyoprotectants such as sucrose or trehalose maintained the physical characteristics, including the diameter and PDI [54]. Considering the stability of CpG ODNs as compared with that of siRNA and mRNA, we will test the stability and immunogenicity of our lyophilized LNPs during the lyophilization-reconstitution process and storage at 4 °C, which may be a priority, as compared with the current unlyophilizable liposome-based adjuvant system (AS01B) for VZV subunit vaccines.

## 5. Conclusions

Overall, LNPs designed for siRNA/mRNA vaccines could efficiently encapsulate gE and nucleic acid immunostimulators, especially CpG ODNs. All of the components, except for Poly I:C (which could be removed), have been approved by the FDA as vaccine components and have shown good safety. These LNP vaccines could induce VZV-gE-specific humoral responses, showing great prospects as varicella vaccines without a potential risk of zoster, and they exhibit potential as zoster vaccines at much lower costs than current vaccines. Theoretically, successful application of these potential LNP-based varicella vaccines may exempt zoster vaccines in the future. In addition, the antigen-specific Th-1 oriented immunogenicity induced by the LNP systems that, described in this study, might highly impact on subunit vaccine developments currently depends on alum adjuvants.

## Figures and Tables

**Table 1:** Designed vaccine composition of each dose.

Vaccine Group	gE (µg)	CpG (µg)		Poly I:C (µg)
		BW006	2395	
Blank				
LNP				
Alum+gE	10			
LNP-gE	10			
LNP-BW006-gE	10	12.5		
LNP-2395-gE	10		12.5	
LNP-PolyI:C-gE	10			12.5
LNP-BW006+2395-gE	10	6.25	6.25	
LNP-BW006+2395+PolyI:C-gE	10	3.125	3.125	3.125

**Table 2:** Vaccine compositions of 20 doses.

Vaccine Group	gE (µg)	CpG (µg)		Poly I:C (µg)
		BW006	2395	
Blank				
LNP				
Alum+gE	200.00			
LNP-gE	299.72			
LNP-BW006-gE	276.09	229.83		
LNP-2395-gE	274.73		297.02	
LNP-PolyI:C-gE	273.37			139.52
LNP-BW006+2395-gE	270.65	149.89	149.89	
LNP-BW006+2395+PolyI:C-gE	250.39	45.23	45.23	45.23
